# Clinical Utilities of Peripheral Blood Gene Expression Profiling in the Management of Cardiac Transplant Patients

**DOI:** 10.1080/15476910701385570

**Published:** 2007-09-04

**Authors:** Kenneth C. Fang

**Affiliations:** XDx, Inc., San Francisco, California

**Keywords:** acute cellular rejection, cardiac allografts, gene expression profiling

## Abstract

Cardiac allografts induce host immune responses that lead to endomyocardial tissue injury and progressive graft dysfunction. Inflammatory cell infiltration and myocyte damage characterize acute cellular rejection (ACR) that presents episodically in either a subclinical or symptom-associated manner. Sampling of the endomyocardium by transvenous biopsy enables pathologic grading using light microscopic criteria to distinguish severity based on the focality or diffuseness of inflammation and associated myocyte injury. Monitoring for ACR utilizes endomyocardial biopsy in conjunction with history and physical examination and assessment of allograft function by echocardiography. However, procedural and interpretive issues limit the diagnostic certainty provided by endomyocardial biopsy. The dynamic profiling of genes expressed by peripheral blood mononuclear cells (PBMCs) enables quantitative assessments of intracellular mRNA whose levels fluctuate during systemic alloimmune responses. Gene expression profiling of PBMCs using a multi-gene ACR classifier enables the AlloMap® molecular expression test to distinguish moderate to severe ACR (*p* = 0.0018) in heart transplant patients. The AlloMap test provides molecular insights into a patient's risk for ACR by distilling the aggregate expression levels of its informative genes into a single score on a scale of 0 to 40. The selection of a score as a threshold value for clinical decision-making is based on its associated negative predictive value (NPV), which ranges from 98 to 99% for values in three post-transplant periods: >2 to ≤6 months, > 6to ≤ 12 months, and >12 months. Scores below the threshold value rule out ACR, while those above suggest increased ACR risk. Incorporating the AlloMap test into immunomonitoring protocols provides an opportunity for clinicians to enhance patient care and to define its role in immunodiagnostic strategies to optimize the clinical outcomes of heart transplant recipients. This summary highlights the concepts presented in an invited presentation at a conference focused on Immunodiagnostics and Immunomonitoring: From Research to Clinic, in San Diego, CA on November 7, 2006.

## INTRODUCTION

**FIG. 1:**
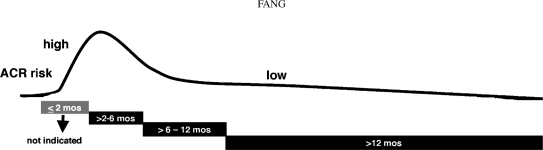
Time-dependent risk and incidence of acute cellular rejection in heart transplant recipients. The risk for acute cellular rejection (ACR) is highest immediately post-operatively, declines during the remainder of the first year, and remains an ongoing clinical concern.

Clinicians caring for cardiac transplant patients must balance the risks and benefits of drug regimens designed to suppress immune responses to the engrafted heart, while also recognizing that acute and chronic forms of rejection may still occur despite good compliance with complex therapies. Heart transplant patients remain at continual risk for the development of acute cellular rejection (ACR), which involves injury to endomyocardial tissue due to inflammatory cell infiltration and myocyte damage that may be accompanied by graft dysfunction or failure (Billingham et al., 1990; Subherwal et al., 2004; Stewart et al., 2005). The incidence of ACR is highest in the immediate post-transplant period and declines during the remainder of the first year and continually thereafter, but remains a clinical concern even many years after transplantation (Figure 1) (Gradek et al., 2001; Patel and Kobashigawa, 2006).

To diagnose ACR, transplant cardiologists perform transvenous biopsy of the right ventricular septum (Caves et al., 1973, 1974), which yields specimens that can be pathologically graded using a scale ranging from normal tissue to severe injury (Billingham et al., 1990; Stewart et al., 2005). The performance of endomyocardial biopsies occurs either as part of surveillance monitoring protocols with procedures scheduled at frequent intervals during the first post-transplant year and intermittently thereafter, or when clinically indicated at any time. Several aspects of the biopsy procedure, including tissue sampling error, invasiveness, patient discomfort, risk of morbidity (Hauck and Edwards, 1992; Baraldi-Junkins et al., 1993; Bhat et al., 1993; Williams et al., 1996; Gradek et al., 2001), and variability in biopsy interpretations (Nielsen et al., 1993; Winters and Mc-Manus, 1996; Marboe et al., 2005), have prompted the development of non-invasive alternatives to enhance immunomonitoring in heart transplant patients (Mehra et al., 2002; Subherwal et al., 2004).

AlloMap® molecular expression testing for the management of acute cellular rejection (ACR) in cardiac allograft recipients (Deng et al., 2006b) exemplifies the clinical utility of gene expression technologies in heart transplant immunodiagnostics and immunomonitoring (Horwitz et al., 2004; Schoels et al., 2004). The administration of immunosuppressive regimens attenuates the immune responses that cause acute and chronic endomyocardial tissue injuries, but requires a delicate vigilance to avoid the immediate and delayed morbidities of either excessive or inadequate therapy. Over-immunosuppression may diminish immune responses and hinder defenses against infectious pathogens or impair tumor surveillance mechanisms, and may also contribute to the development of metabolic complications or end-organ toxicities. Under-immunosuppression enhances a patient's risk for ACR and allograft dysfunction or failure. By profiling the expression of a discrete cohort of genes correlating specifically with ACR, the AlloMap test offers an alternative, non-invasive approach for immunomonitoring based on the analysis of circulating immune effector cells in peripheral blood samples of heart transplant patients (Deng et al., 2006b).

Quantitative measurements of intracellular mRNA levels in peripheral blood mononuclear cells (PBMCs) using the real-time polymerase chain reaction (rt-PCR) enables the AlloMap test to distinguish dynamic changes in the expression of genes correlating with the absence or presence of acute cellular rejection (ACR) (*p* = 0.0018) in cardiac allografts. The multi-gene ACR classifier provides molecular insights into a patient's risk for ACR by distilling the cumulative expression levels of its informative genes into a single score on a scale of 0 to 40 by incorporating weighted, gene-specific coefficients derived during the classifier's development and then validated in the CARGO Study (Deng et al., 2006b). The clinical utility of the AlloMap test derives from its high negative predictive value (NPV), enabling it to guide clinicians in ruling out current ACR in the context of the time elapsed since transplantation (time post-transplant) (Deng et al., 2006b; Mehra et al., 2006). Although the incidence of ACR is highest in the immediate peri-operative period, rates decline during the first year and thereafter (Eisen et al., 2003; Klingenberg et al., 2003). However, ACR also occurs in an episodic manner with variable or progressive severity that necessitates diagnostic protocols that include both surveillance and symptom-driven immunomonitoring (Mehra et al., 2002; Patel and Kobashigawa, 2006). The inclusion of the AlloMap test in such protocols may facilitate convenient and repetitive diagnostic testing in cardiac transplant recipients as its performance requires a peripheral blood sample rather than an invasive biopsy procedure.

### Cardiac Allograft Pathology

Host immune responses to cardiac allografts induce inflammatory cell infiltration in the endomyocardium, resulting in a spectrum of tissue injury that may be accompanied by myocyte damage and dysfunction of the engrafted heart. Monitoring for subclinical and clinically suspected episodes of ACR includes routine history and physical examinations supplemented by serial functional assessments by echocardiography and histopathologic evaluations of endomyocardium sampled by transvenous biopsy of the right ventricular septum. Patterns of cellular infiltration and myocyte damage observed using light microscopy enable pathologists to assign grades along a spectrum from normal endomyocardium to severe ACR using the 1990 (0, 1A, 1B, 2, 3A, 3B or 4) or 2004 (0R, 1R, 2R or 3R) systems approved by the International Society of Heart and Lung Transplantation (ISHLT) (Billingham et al., 1990; Stewart et al., 2005). Moderate to severe grades of ACR (≥3A or ≥2R) define clinical thresholds for augmented immunosuppression therapy (Kobashigawa et al., 2006) to attenuate alloimmune responses with the goals of minimizing tissue injury and preserving allograft function.

The limitations of tissue-based ACR diagnosis associated with the invasive biopsy procedure include inadequate tissue samples (due to heterogeneity of endomyocardial injury), obscured histopathology (e.g., septal fibrosis), venous access difficulties, and risks of morbidity such as tricuspid valve insufficiency, dysrhythmias or ventricular perforation (Hauck and Edwards, 1992; Baraldi-Junkins et al., 1993; Bhat et al., 1993; Williams et al., 1996; Gradek et al., 2001). The pathologic interpretation of biopsy specimens may also yield diagnostic uncertainty due to intra- and inter-reader variabilities (Nielsen et al., 1993; Winters and McManus, 1996; Marboe et al., 2005) and the occurrence of nodular endocardial infiltrates known as Quilty lesions (Pardo-Mindan and Lozano, 1991; Joshi et al., 1995; Marboe et al., 2005). Despite such limitations, endomyocardial biopsies remain a familiar diagnostic tool that may be complemented by new molecular insights provided by the AlloMap test.

### AlloMap Genes

Genes comprising the AlloMap test have been implicated in diverse immunoregulatory pathways in a variety of immune and non-immune cells (Deng et al., 2006b; Zeevi et al., 2006). The development of the ACR classifier emphasized an inclusive strategy with candidate gene biomarkers for ACR identified either by implication in molecular pathways substantiated in the literature or through a discovery process utilizing leukocyte-specific gene microarrays. The subsequent validation of these biomarkers using quantitative rt-PCR yielded a subgroup of genes individually correlating with the clinical endpoint of ACR. Included in this subgroup were genes already implicated in a myriad of alloimmune responses detected by genomic methods in engrafted tissues or other clinical specimens from both cardiac and non-cardiac allograft recipients. However, the linear discriminant analysis used to derive the classifier identified a combination of 20 genes that best distinguished cardiac allograft ACR, with a subgroup of 11 informative genes that did not include many previously identified in the literature (Deng et al., 2006a; Halloran et al., 2006).

The remaining 9 AlloMap genes serve as control or reference genes used for test accuracy and reproducibility (Deng et al., 2006b). The 11 informative genes correlating with ACR are PDCD1, SEMA7A, RHOU, MARCH8 (MIR), WDR40, ITGAM, IL1R2, FLT3, (C6orf25) G6B, PF4 and ITGA4, suggesting transcriptional influences by T-cells, B-cells, neutrophils, platelets, and hematopoietic progenitor cells in a variety of pathways regulating cell activation or homeostasis, including 3 genes (IL1R2, ITGAM and FLT3) associated with corticosteroid-sensitive mechanisms (Deng et al., 2006b).

**FIG. 2:**
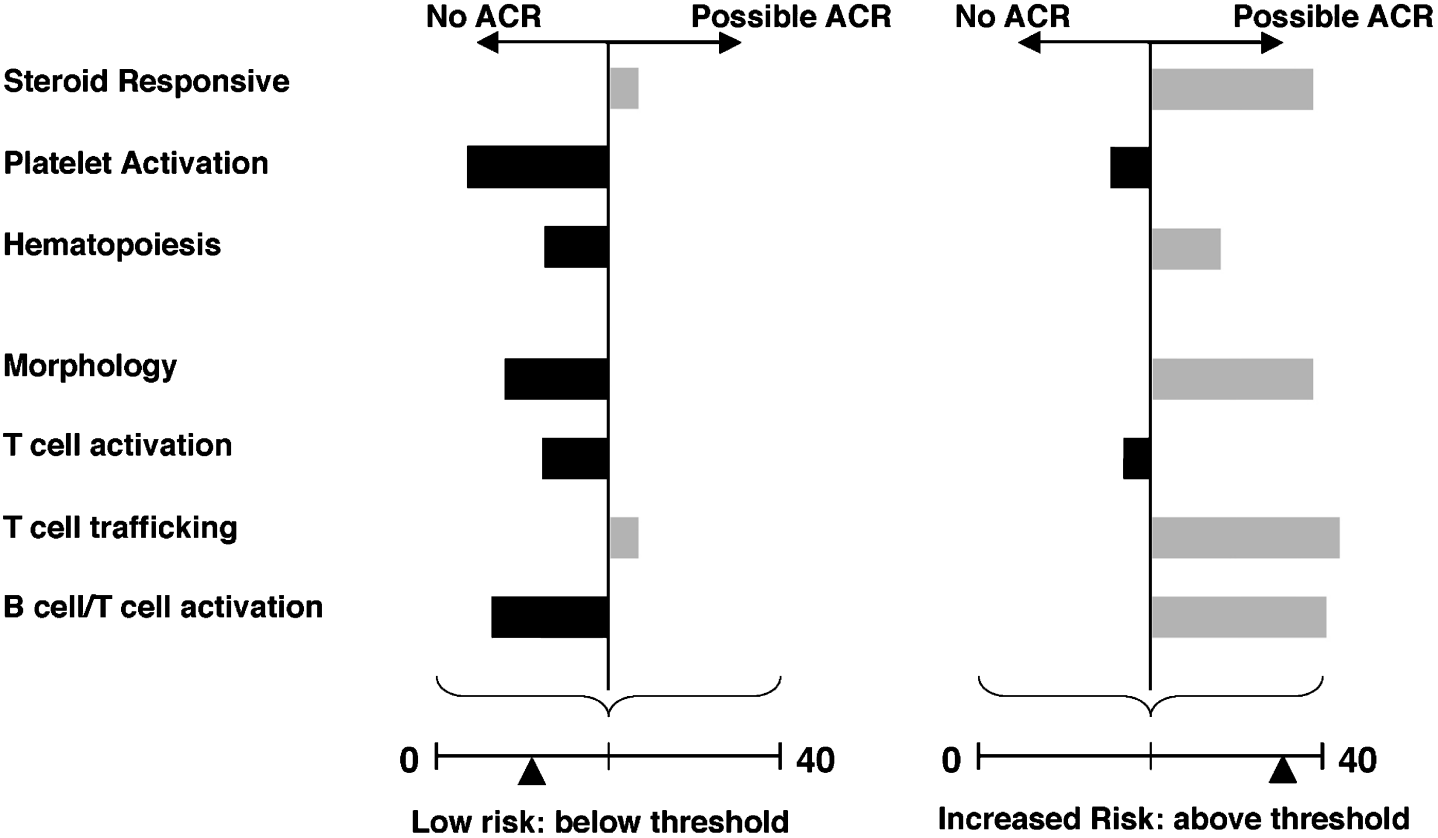
The AlloMap test score reflects differential gene expression in diverse regulatory pathways. The AlloMap test classifier for acute cellular rejection (ACR) consists of 20 genes, including 11 that inform on a variety of regulatory pathways and contribute to its 7 terms, which correspond to the expression of individual genes or the average expression levels of coordinately expressed genes termed metagenes. The classifier also includes 9 genes used for normalization of gene expression and quality control. Low AlloMap scores on a scale from 0 to 40, below the clinician-specified threshold, can be used to rule out ACR. Up-regulated (gray) and down-regulated (black) expression of genes, as assayed in peripheral blood mononuclear cells (PBMCs) by qPCR, contributes to the AlloMap score.

AlloMap scores derived using the ACR classifier reflect the summation of all individual cycle threshold (C_t_) values measured by rt-PCR with relative weighting determined by each gene's accompanying coefficient (Figure 2) (Deng et al., 2006b). The inclusion of PDCD1 highlights its importance in T-cell stimulatory pathways in mediating ACR, as other genes also implicated in such mechanisms did not demonstrate statistical performance or reproducibility sufficient for their inclusion in the assay panel (Deng et al., 2006a; Halloran et al., 2006). Both the molecular mechanisms regulating these dynamic expression patterns and the associations of the test's genes with host responses to cardiac allografts remain an ongoing focus of investigation.

The issue of the physiologic relevance of individual gene signatures is one of a number of concerns raised about the process for developing clinically useful molecular classifiers. Additional concerns have focused on the applicability of statistically significant study results to the broad clinical population, the validity of reference standards used as clinical endpoints (e.g., biopsy grades), and the balance between prioritizing the robust performance of a multi-gene algorithm and the potential cost of excluding a single gene previously shown to have mechanistic relevance (Halloran et al., 2006). The development process of the AlloMap test addressed many of these issues (Deng et al., 2006a), which are clarified in detail in this work. Ongoing research in translational genomics will likely assuage many of these concerns and reveal further clinical benefits of the synergy of integrating genomic information, clinical data, bioinformatics and statistics in the development of molecular diagnostic assays.

### AlloMap Score Interpretation

**FIG. 3:**
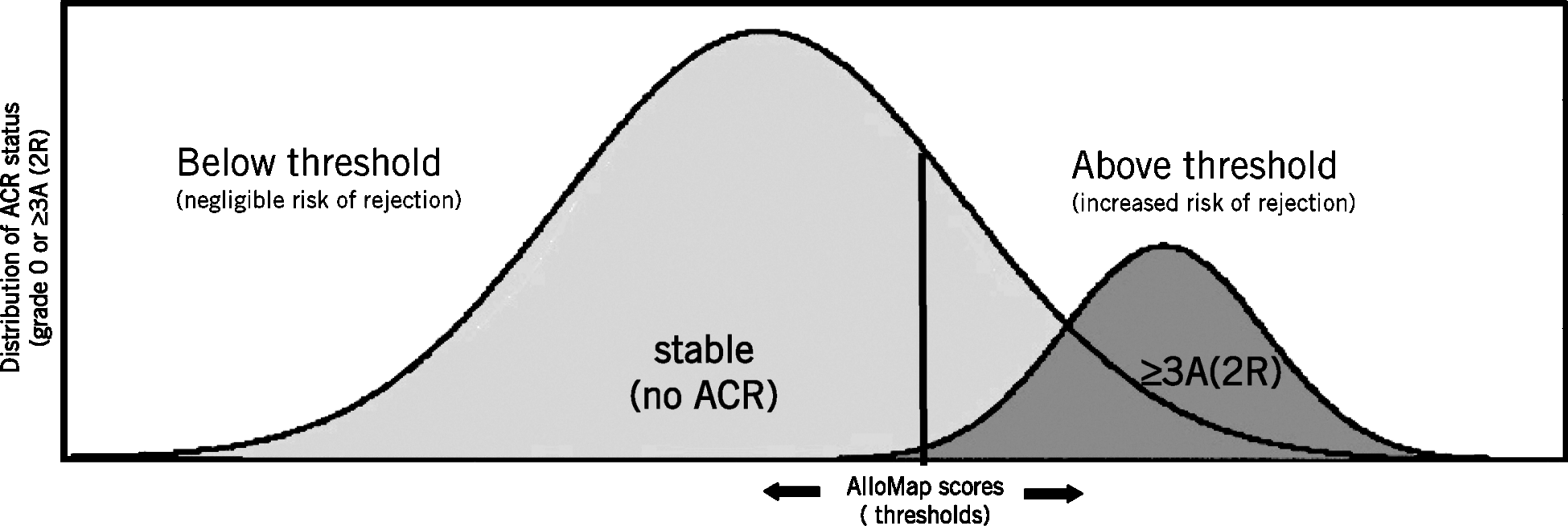
Interpretation of the AlloMap test score depends on a clinically specified threshold. The clinician interprets the AlloMap score relative to a pre-selected threshold on the 0-to-40 scale and the associated negative predictive value. A score below the threshold indicates a low risk for acute cellular rejection (ACR), thus ruling out ACR. A score above the threshold suggests an increased risk for moderate-to-severe (≥3A/2R) ACR. By understanding the patient's clinical course, the time-dependent ACR risk, the clinical tolerance for false-negative or false-positive results, and practice-specific demographics, clinicians may personalize the management of heart transplant patients through the selection of the AlloMap threshold.

The clinician assesses a patient's ACR risk by interpreting the AlloMap score relative to a pre-selected threshold value on the 0-to-40 scale and its associated NPV. Scores below the chosen threshold indicate a low ACR risk, thus ruling out ACR, while those above suggest an increased risk for ACR. Figure 3 illustrates the decision analysis faced by the clinician in the selection of a threshold for a given patient. Since the high NPV enables an AlloMap score to rule out grade ≥3A rejection, the objective is to choose a threshold value that enables an individual (below threshold) AlloMap score to rule out grade ≥3A rejection (e.g., a true negative result), while minimizing the likelihood that an above-threshold score might be associated with no ACR (i.e., a false positive result). Selecting progressively lower threshold values (leftward movement of the threshold bar) may assure that an individual score result correlates with no ACR, however, at the cost of an increased likelihood of a false positive result with an above threshold score.

By contrast, choosing higher threshold values (rightward movement of the threshold bar) may minimize the likelihood of false positive results, but increases the likelihood that a grade 3A rejection may be present with a below threshold score (i.e., false negative result). As with all diagnostic tests, the clinician must perform a preemptive risk-benefit analysis that considers the potential consequences of any false negative or positive result for a given patient's clinical status.

The time post-transplant (based on the date of testing relative to the transplant date) guides selection of the threshold in each of three time intervals (>2to ≥6 months, >6to ≥12 months, or >12 months) that reflects the appropriate time-dependent risk for ACR. The initiation of AlloMap testing in stable patients at >2 months post-transplant is based on the rationale that the post-operative clinical course of most patients becomes relatively stable after 2 months. The NPV is the test performance parameter that guides the interpretation of AlloMap scores in each time period; based on the prevalence of ACR in a given population, it indicates the ability of a negative test result to predict the absence of grade ≥3A ACR (true negative) in contrast to instances when grade ≥3A ACR is actually present (false negative) (Riegelman, 2005b). The time-dependent prevalence of grade ≥3A ACR for patients enrolled in the CARGO Study permitted the determination of an NPV for every AlloMap score in each of the three time periods (Deng et al., 2006b).

Since the NPV indicates the percentage of test results that correctly predicts the absence of grade ≥3A ACR, a high NPV is desirable to rule out ACR. To highlight the importance of the NPV, the following example illustrates the interpretation strategy for an individual AlloMap score based on the selection of 34 as the threshold value for a patient evaluated at one year post-transplant. For this threshold and time post-transplant, the NPV of 99.2% indicates that 99.2% of all results lower than 34 will successfully predict the absence of ACR (true negative). By contrast, 0.8% of results below the threshold may yield a false negative result, indicating that in 8 instances for every 1000 test events a score below 34 may be associated with the presence of ACR. Therefore, determining the time post-transplant for the patient at each test date and considering the NPV with its estimates of true negative and false negative results both play important roles in guiding the clinician in selecting the appropriate threshold value.

### Threshold Selection

The selection of a threshold value depends not only on the time post-transplant, but also on the clinician's knowledge of a patient's post-operative course and the prevailing practice environment. Inherent in any test interpretation is the risk analysis associated with the balancing of false negative and false positive test results. A false negative result indicates a missed diagnosis of a disease or condition, with the potential clinical consequences determined by the magnitude of the associated morbidities or sequelae. A false positive result may adversely influence clinical decision-making by prompting an action such as treatment or a procedure that is unnecessary given the true absence of the disease or condition (Riegelman, 2005a). The ability of a patient to tolerate a clinical action based on a false negative or false positive result depends on multiple factors, including the frequency or prevalence of the disease, the current status, and the prior course, with any clinical instability lowering the tolerance for errant diagnoses. If the disease prevalence is relatively low or a false negative result may be clinically tolerable, then a higher AlloMap threshold may be appropriate.

By contrast, a lower AlloMap threshold may be optimal if the disease prevalence is comparatively high or if a false negative result is likely to contribute to adverse outcomes. Factors affecting threshold selection include not only the time-dependent disease prevalence, but also the experience or management style of clinicians and the distribution of patient risk profiles in a given practice. Therefore, the consideration of a patient's course, the disease prevalence, the clinical tolerance for false negative or false positive results, and practice-specific demographics is likely to guide the clinician's approach to personalizing the care of a heart transplant patient through the selection of the AlloMap threshold.

### Clinical Experience with AlloMap Testing

Interpreting the AlloMap score initially in the context of biopsy data enables clinicians to use their accrued experience with pathology as they incorporate molecular expression testing into their management protocols. In the CARGO Study biopsy grades served as a surrogate for alloimmune status in both the development and verification of the AlloMap test, and also enabled the derivation of its performance parameters based on the observed agreement of their results (Deng et al., 2006b). However, disagreement between biopsy and AlloMap results, or discordance, is also expected as each approach uses different, but complementary technologies to assess ACR.

An accurate biopsy grade requires the satisfaction of criteria for tissue sample adequacy and tissue injury based on light microscopic criteria assessing inflammatory cell infiltration, myocyte damage, and other features distorting normal endomyocardial architecture (Billingham et al., 1990; Stewart et al., 2005). The AlloMap test score results from the quantification of intra-cellular mRNA levels of 20 genes, which in combination differentiate the absence or presence of ACR in cardiac allografts (Deng et al., 2006b). An initial multi-center experience with the clinical adoption of AlloMap testing in conjunction with biopsy procedures illustrated four scenarios in which the results of both tests either demonstrate diagnostic agreement (concordance) or disagreement (discordance).

The concurrent performance of endomyocardial biopsy and AlloMap testing in 211 evaluations of patients at or beyond 1 year post-transplant enabled the calculation of agreement or disagreement rates between negative (>3A/2R) or positive (≥3A/2R) biopsy results and negative (below threshold) or positive (above threshold) AlloMap scores. An AlloMap score below the threshold value of 34 accompanied a negative biopsy result in 69.7% of the test events, while an above threshold score occurred with all (n = 5) positive biopsies. No instances of a positive biopsy occurred with a below threshold AlloMap score. By contrast, an above threshold AlloMap score (positive result) accompanied 59 biopsies interpreted as negative (<3A/2R), yielding a discordance rate of 28%. In this analysis using a threshold value of 34 at 1 year post-transplant, the derived performance parameters for the AlloMap test included an NPV of 100% and a PPV of 7.8% (Starling et al., 2006). These data substantiate those yielded by the CARGO Study and support the use of the AlloMap test in ruling out ACR.

The expectation of discordance between biopsy grades and AlloMap scores stems from an analysis of established and postulated factors that may influence the false negative or false positive rates of their respective results. Interpretation of endomyocardial biopsies by a participating center's pathologist is the standard utilized to assess the clinical endpoint of ACR in clinical studies, which may also require re-interpretation of the original biopsies by a panel of study pathologists as part of the study design (Nielsen et al., 1993; Winters and McManus, 1996; Marboe et al., 2005). The early demonstration of the ability of transvenous endomyocardial biopsy to obtain tissue to assess ACR (Caves et al., 1973, 1974) led to the development of consensus criteria for its interpretation to facilitate grading uniformity and communication, as stipulated in the original 1990 grading system that underwent revision in 2004.

Both grading systems emphasize the sufficiency of tissue samples for analysis and the need to adhere to criteria delineating the following: the focality or diffuseness of inflammatory cell infiltration, any evidence of myocyte damage, the presence of nodular endocardial infiltrates known as Quilty lesions, and additional morphologic criteria associated with antibody-mediated rejection (AMR) (Billingham et al., 1990; Stewart et al., 2005). Therefore, clinically indeterminate biopsy results may result due to issues associated with either the procedure or tissue interpretation. Sampling error may result from the acquisition of tissue that is of insufficient quantity for analysis, not representative of the heterogeneous injury, or obscured by scarring. Known variabilities in endomyocardial biopsy interpretation may also contribute to diagnostic uncertainty in both practice and investigational settings.

### Endomyocardial Biopsy Interpretation

Both intra- and inter-reader variability of endomyocardial biopsies occurs, due in part to potentially confounding Quilty lesions, which has led to the requirement for biopsy re-interpretation and grade re-assignments to enhance the stringency of the clinico-pathologic ACR endpoint in studies (Nielsen et al., 1993; Winters and McManus, 1996; Deng et al., 2006b). An analysis of pathology data in the CARGO Study substantiated the known interpretive variability for biopsies and also demonstrated that individual pathologists may favor the assignment of moderate to severe ACR grades. Of all biopsies graded as ≥3A by study center pathologists, 40% were confirmed by a 3-member pathology panel, while the best concordance for assigning ≥3A grades among the three panel pathologists was 58% (Marboe et al., 2005). These biopsies with ≥3A grades confirmed by the panel were used to develop and validate the AlloMap test, with the calculation of an NPV for each score based on the time-dependent ACR prevalence.

The AlloMap test has a high NPV, ranging from ∼98 to 99%, for scores considered as appropriate clinical threshold values in each of the three time periods post-transplant (Deng et al., 2006b). In addition to the molecular and bioinformatic strategies used to derive the classifier, the high NPV of the AlloMap test also stems, in part, from the current lower prevalence of ACR resulting from state-of-the-art immunosuppression and monitoring strategies (Eisen et al., 2003; Subherwal et al., 2004; Patel and Kobashigawa, 2006). The associated positive predictive value (PPV) for individual AlloMap scores is less than 15%, due mainly to the relatively low time-dependent prevalence of grade ≥3A ACR (Gradek et al., 2001; Subherwal et al., 2004), highlighting the ability of disease prevalence to limit a test's PPV (Riegelman, 2005b). Thus, while the AlloMap test may guide clinician's in ruling out ACR, it is limited in its ability to rule in ACR.

Based on the hypothesis that genes in the ACR classifier may assess molecular pathways active in other alloimmune processes, investigators have postulated that above threshold AlloMap scores may also be manifestations of cardiac allograft vasculopathy (CAV) (Yamani et al., 2005) or AMR (Starling et al., 2006). In addition, the sensitivity of rt-PCR for quantifying diverse intracellular genes in PBMCs also suggests that the AlloMap test may assess early, systemic alloimmune responses that contribute to ACR prior to the development of tissue manifestations that suffice for detection by light microscopy and satisfy the ISHLT grading system criteria.

### Negative Biopsy and Above-Threshold AlloMap Results

Discordance highlighted by a negative biopsy result and a “high” (above threshold or positive) AlloMap score may be clarified by reviewing the diverse factors influencing the relative likelihood of either result being a false negative or false positive. In the CARGO Study a biopsy assigned grades 0 (n = 416), 1A/1B (n = 174) or 2 (n = 159) by a study center pathologist received confirmation by the 3-member pathology panel in 87%, 48% or 17% of cases, respectively; the agreement of the 3-member panel on their own assignments of grades 0, 1A/1B or 2 for these biopsies was 93%, 71% or 60%, respectively (Marboe et al., 2005). The observed interpretive variability for these grades that are used to define a negative biopsy (<3A) suggests some diagnostic uncertainty and highlights the potential for a false negative biopsy result. Although above threshold AlloMap scores suggest increased risk for ACR, investigators have postulated that other factors may also yield “high” scores.

The maximum PPV of AlloMap threshold values ranges from 14.3% (>2 to ≤6 months) to 9.9% (>12 months), demonstrating the influence of the diminishing time-dependent prevalence of ACR and that all above threshold test scores do not indicate an episode of ACR. Some investigators have hypothesized that “high” AlloMap scores later post-transplant may be associated with the development of CAV (Yamani et al., 2005). Data from the CARGO Study showed that above threshold AlloMap scores may also represent grade 1B biopsies, as the mean score for this subgroup of mild ACR is indistinguishable from that for grades ≥3A (Bernstein et al., 2005, 2007), which suggests the molecular similarity of these grades. The validation of these hypotheses and others postulating early sensitivity of the AlloMap test for systemic alloimmune activation or AMR remain the focus of ongoing investigations.

The potential also exists for discordance generated by a positive (≥3A/2R) biopsy result and a “low” (below threshold or negative) AlloMap score. In addition to the known intra- and inter-reader variability in interpreting ≥3A/2R results that can result in false-positive biopsy results (Marboe et al., 2005), other data suggest that some biopsies graded as ≥3A may resolve without therapy (Klingenberg et al., 2003). An as yet unproven hypothesis for a below threshold AlloMap score is that in this setting it may reflect resolving systemic alloimmune activation prior to the restoration of normal tissue architecture. Since the AlloMap test does reflect a patient's alloimmune status, an additional factor that may yield a lower than expected score is either intended or unintended augmentation of steroid immunosuppressive therapy and its relationship to the timing of the acquisition of the blood sample for testing.

The management of discordant clinical data often includes an assessment of the certainty or robustness of the information provided by a given diagnostic modality. Since the differential diagnosis for potentially discordant results between biopsy grades and AlloMap scores is broad, avoiding (or minimizing) discordance might depend on enhancing the information provided by biopsies or improving molecular understanding of transplant-associated genomic responses. The revision of the classification system for acute cellular rejection represents one collaborative effort between clinicians and pathologists to improve the utility of the biopsy grades (Stewart et al., 2005). The application of genomic technologies to clinical disorders has been explosive and is likely to clarify further the molecular mechanisms mediating recipient responses to cardiac allografts. Additional genomic information may help clarify any discordance that may arise in comparing the results of molecular diagnostic assays to more traditional tests.

### Potential Clinical Influences on AlloMap Score Interpretation

Investigations of potentially confounding influences on the AlloMap score and its interpretation have focused on factors that directly or indirectly affect transplant-associated immunoregulatory pathways. During the early period after transplantation, clinicians maintain a posture of vigilance for infection in the setting of the diminishing effects of induction therapy and decreasing steroid immunosuppression. Although monoclonal or polyclonal antibody therapeutics target circulating immune effector cells, an analysis of the CARGO Study data identified neither an influence of induction therapy on the AlloMap score, nor any significant changes in the expression level of individual constituent genes (Mehra et al., 2007).

The objective of minimizing corticosteroid side effects has led to the practice of steroid withdrawal, with doses often decreased to below 20 mg daily by 1 to 2 months post-transplant (Eisen et al., 2003; Rosenbaum et al., 2006). In this dose range, corticosteroids do not influence the AlloMap score, but doses exceeding 20 mg daily may alter the expression of three ACR classifier genes, including IL1R2, ITGAM and FLT3, that may contribute to a lower AlloMap score (Deng et al., 2006b; Starling et al., 2006). Therefore, the usual practice of maintaining low oral doses mitigates the effects of steroids on patient selection for AlloMap testing. Although investigations of other immunosuppressive agents in the CARGO Study have not identified any influences on the AlloMap test, their potential effects on gene expression remain a focus of study.

A cost of immunosuppression is increased patient susceptibility to opportunistic infections, and more frequent or severe sequelae from bacterial infections (Fishman and Rubin, 1998). The potential overlap between innate (infection-associated) and adaptive (transplant-associated) immune responses in cardiac allograft recipients has also engendered concerns about the potential effects of infection on AlloMap test interpretation. Given the clinical impact of cytomegalovirus (CMV) infection or disease (Zamora et al., 2005), an analysis of the CARGO Study data investigated its influence on PBMC gene expression profiles and demonstrated that its peripheral molecular signature did not overlap with genes comprising the AlloMap test; moreover, the absence or presence of CMV infection did not influence AlloMap test scores in patients with ACR (Deng et al., 2004). To assess the broader influence of infection, the AlloMap genes were compared to those identified in host responses to bacterial, fungal and viral pathogens; these analyses did not reveal any overlap between the ACR classifier genes and those identified in infection response pathways in various studies at the level of cells, animal models, or clinical investigations (Jenner and Young, 2005).

Based on the design of gene expression analyses in the CARGO Study, the likelihood that either immunosuppression therapy or infection might influence the AlloMap test interpretation is also diminished by the inclusion of patients with these clinical phenotypes in both the experimental and control groups of the studies performed (Deng et al., 2004). Since gene profiling analyses distinguish patterns of expression based on differences in the clinical phenotypes of the patients, the inclusion of these clinical influences in both study groups suggests that any potential influences on gene expression would be minimized.

### AlloMap Testing and Clinical Protocols

The incorporation of the AlloMap test into management protocols will enable clinicians to assess its utility and to answer many questions raised by the technology and sensitivity of molecular diagnostics. Issues of timing, frequency and efficacy already familiar to clinicians using other diagnostic tests will also guide the adoption of the AlloMap test. Consideration of these factors creates an opportunity to personalize a patient's care and optimize a transplant center's clinical outcomes. A patient's course with respect to ACR episodes guides ongoing diagnostic monitoring and therapeutic management for individuals, while the characterization of risk profiles identifies patient subgroups requiring heightened attention and appropriate tailoring of established practices.

As with all diagnostic tests, the interpretation of an individual AlloMap test result is optimal in a longitudinal fashion (Starling et al., 2006) that permits interpretation of a current result in the context of the patient's clinical baseline to identify deviations. The concurrent performance of endomyocardial biopsy and AlloMap testing may be useful to establish an initial baseline, which then enables interpretation of subsequent below or above threshold scores by comparison to prior scores, either with or without additional biopsy data. Information provided by the history and physical examination, echocardiography and other adjunctive diagnostic testing provide additional context, with the objective insights supplied by AlloMap scores supplementing the clinical acumen used in ACR risk assessments.

## SUMMARY

AlloMap molecular expression testing is a clinical application of gene expression profiling that enables a clinician to rule out ACR in cardiac allograft recipients. Each AlloMap threshold value has an associated NPV that guides a clinician in selecting the appropriate threshold for each patient in three post-transplant periods, including >2 to ≤6 months, >6 to ≤12 months, and >12 months. An AlloMap score below the threshold rules out ACR, while one above threshold suggests increased ACR risk. Early clinical experience based on concurrent endomyocardial biopsy and AlloMap testing substantiates the utility of the test in ruling out ACR and also highlights expectations of test result discordance, which occurs more commonly with a negative biopsy and a “high” (above threshold or positive) AlloMap score.

The consideration of factors contributing to false negatives or false positives will facilitate the interpretation of both tests' results and subsequent decision analyses. The sensitivity of genomic technologies used in the development of the AlloMap test and future molecular diagnostic tests will likely require a collaborative approach between industry and academic investigators to explore and answer the many provocative questions. Incorporation of the AlloMap test into management protocols will enable clinicians not only to assess its clinical utility further, but also to define its role in strategies optimizing the clinical outcomes for heart transplant patients.

## Abbreviations

ACR: acute cellular rejection
AMR: antibody-mediated rejection
CARGO: Cardiac Allograft Rejection Gene Expression Observational
CAV: cardiac allograft vasculopathy
CMV: cytomegalovirus
ISHLT: International Society of Heart and Lung Transplantation
NPV: negative predictive value
PBMC: peripheral blood mononuclear cell
PPV: positive predictive value
rt-PCR: real-time polymerase chain reaction
